# Exploring the Application and Optimization Strategy of the LMBP Algorithm in Supply Chain Performance Evaluation

**DOI:** 10.1155/2022/7977335

**Published:** 2022-08-23

**Authors:** Fei Gu

**Affiliations:** Capital University of Economics and Business, Beijing 100070, China

## Abstract

In recent years, with the emergence of new technologies, big data, artificial intelligence, and other technologies have had a greater impact on supply chain management. Among them, big data analysis capability, as one of the important capabilities that supply chain enterprises should have, has a particularly significant impact on supply chain resilience management. From the perspective of performance management, based on supply chain resilience theory, the relationship between the supply chain performance management level, supply chain collaboration, and other supply chain resilience elements, as well as big data analysis capability and supply chain performance can be analyzed to study the impact of big data analysis capability on supply chain performance of enterprises of different scales. The impact on the level of supply chain performance is being studied. This paper investigates the problem of supply chain performance evaluation and optimization based on the LMBP algorithm and provides some references for supply chain performance evaluation and optimization.

## 1. Introduction

Since the reform and opening up, China's logistics industry has gone through the stages of learning, introducing, learning from, and independent innovation, promoting China's transformation from a weak logistics country to a large logistics country and a strong logistics country, and is developing into a world supply chain innovation center, which is another major “windfall” for China's economic and social transformation and upgrading. In this process, along with the development of big data, artificial intelligence, and other emerging technologies, the daily operation and management of the supply chain are facing great challenges and must make timely responses to the rapid changes in the market environment [1, 2]. Big data technology is an effective information management technology that plays a huge role in business operation and management. In a complex business environment, various supply chain risks can cause supply chain disturbances or even disruptions. In order to cope with various risks, enterprises will use the function of big data analysis and prediction, prompting big data technology to play a role in business analysis to assist in decision making, improve the level of supply chain performance, and achieve better competitive results. However, how big data analytics works for companies of different sizes, what impact it has on supply chain performance, and how important it is have not been explored in depth. In addition, from the perspective of performance management, supply chain resilience management involves different aspects of supply chain management, and it is necessary to study how different elements are embedded in big data technology to play a role. To this end, this paper explores the problem of supply chain performance evaluation and optimization based on the LMBP algorithm, with a view to explore the path and direction of sustainable supply chain operation from the perspective of supply chain performance evaluation.

## 2. Basic Theory of Supply Chain Performance Evaluation

### 2.1. Basic Connotation of Supply Chain Performance

#### 2.1.1. The Concept of Performance

In 1997, Bititci et al. argued that performance evaluation is the process of determining the extent to which an organization or individual achieves its goals and strategies [3]. In this paper, the view of performance evaluation is that the process of appraisal focuses on output performance, i.e., the results of work, but at the same time, in the process of supply chain performance improvement, more attention is paid to the input elements, i.e., the impact of different actions on output results so that the overall level of supply chain performance can be adjusted and optimized immediately.

#### 2.1.2. Concept of Supply Chain Performance

A supply chain is a network chain structure, which is composed of several participating node enterprises [4]. Supply chain performance is the common behavior of all participants in this chain structure and the final result achieved, and it also refers to the unity of effectiveness and efficiency in the process of supply chain operation. Therefore, the supply chain performance should be examined not only in terms of the final efficiency of its input costs but also the effectiveness of the supply chain output, i.e., whether the needs of end customers are met, and how reliable, flexible, and timely the overall operation of the supply chain is, all of which are considered indicators of effectiveness. In this paper, both efficiency and effectiveness are taken into account in evaluating supply chain performance.

### 2.2. Related Research

#### 2.2.1. Supply Chain Research Combined with the Bionic Algorithm

A bionic algorithm is a computer technology that simulates the biological evolution process with the characteristics of self-organization, self-adaptation, continuous solution optimization, and search. Among them, the neural network algorithm is one of the most widely used methods. By analyzing and training a large amount of historical data, the neural network algorithm (NNA) compares the system training output results with the target output results and repeatedly corrects the neural network until the mean square error reaches the set range [5–7]. The supply chain performance evaluation model established according to this method is usually able to predict the supply chain performance status within a certain period of time but requires as much historical data as possible, including both the original index data set (usually data of each evaluation index) and the target value data set (usually the performance level).

#### 2.2.2. Evaluation of Supply Chain Performance in Apparel Field

In the field of apparel, where this paper is based, Jiang et al. analyzed and optimized the supply chain of China's apparel industry in order to improve the response speed of enterprises to the market and enhance the core competitiveness of the apparel industry. Shao Dan et al. used the Balanced Score Card (BSC) index system, applied the hierarchical analysis method to determine the index weights, and used the fuzzy comprehensive evaluation method to establish an evaluation model for performance evaluation of hypermarket apparel retailing, as well as developed and designed the corresponding evaluation software package according to the model idea [8–10]. Zhao analyzed and discussed the design of KPI indicators for sales staff of a garment company in different development stages of the company and made clear suggestions on the direction of efforts and work focus of the company's sales staff. Li Min et al. construct a comprehensive strength evaluation system for apparel fabric suppliers, build a multiobjective model with the three most important factors of quality, price, and delivery as the target criteria, classify suppliers into four types such as strategic, potential, leverage, and bottleneck, and select three of them as case companies. Take the decision problem of purchasing 20,000 meters of a certain fabric as an example, scientifically allocate fabric purchasing volume, and propose corresponding purchasing strategies.

Based on the above research findings, this paper selects the LMBP (Levenberg–Marquardt Back Propagation) algorithm of the feedback neural network as the theoretical basis, combines the current situation of the enterprise of example A, reasonably determines the evaluation indexes and weights, and proposes several optimization schemes based on the analysis results of training data.

## 3. Research Model

### 3.1. Principle of the LMBP Algorithm

**Figure 1:**
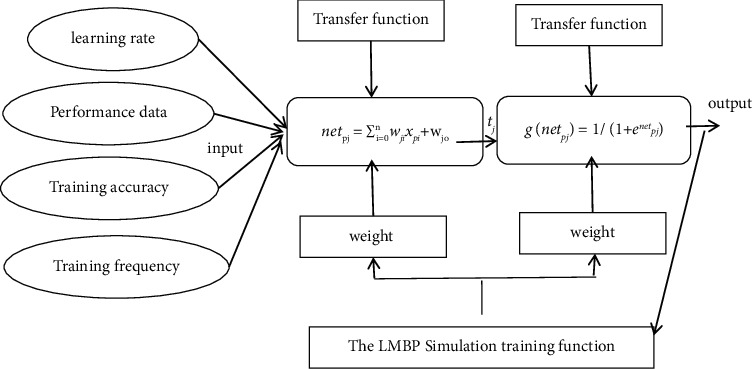
Neural network of the LMBP algorithm based on supply chain performance evaluation.

As one of the feedback neural network algorithms, the LMBP algorithm can continuously modify the network structure and optimally adjust the weights and transfer functions between the levels.  Figure 1 shows the neural network of the LMBP algorithm based on supply chain performance evaluation. The iterative process of the algorithm follows the following steps:

- Initialize the weight values *w* and *μ*, and *μ* usually takes the value 0.01 [11].
- Calculate the mean square error sum of all inputs, *E*(*w*)=*e*^*T*^*e*, where *W*=[*w*_1_, *w*_2_,…, *w*_*N*_] , *e* is the error vector.
- Solve Δ*W*_*kj*_=−[*H*+*μl*]^−1^*J*^*T*^*e* to obtain the weight vector Δ*w*=−[*J*^*T*^*J*+*μl*]^−1^*J*^*T*^*e* , where *J* is the Jacobian matrix, *μ* is the learning rate, and *β* is affected by the results (0 < *β* < 1).
- Repeat the calculation of *E*(*w*) . Here, *e* is the error vector; *μ* is the variable controlling the learning process; *H*=*J*^*T*^*J* is the approximate Hessian matrix.

Based on the above principles, the neural network model of the LMBP algorithm based on supply chain performance evaluation is constructed.

### 3.2. Evaluation Indexes and Weights

Enterprise A, founded in 1996, is a diversified business brand enterprise group company mainly engaged in apparel manufacturing and terminal retailing and has been listed on the National Small and Medium Enterprise Stock Transfer System Limited Liability Company (New Third Board) [12, 13]. Through combing the company's financial report and field research from April 2019 to May 2020, we learned that the main business of enterprise A includes the design and development, brand promotion, and terminal retail of its own brand of men's formal wear and the manufacturing and terminal retail of its own brand professional wear. The company has made the leap from product operation to brand building through four periods since its construction and has now entered the brand maturity period.

Based on the performance evaluation theory, the needs of the apparel industry, the characteristics of the time, and the actual data of the case company, five basic criteria for evaluation were first selected, including cost, time, quality, reliability, and flexibility. The secondary evaluation indicators were selected using frequency analysis and then selected through a consulting method, i.e., the 21 primary indicators were scored on a 5-segment scale by the core management of Company A based on their level of importance. “Very unimportant,” “relatively unimportant,” “average,” “relatively important,” and “very important” were scored as 1–5, respectively, so as to measure the ratings and suggestions of the actual managers and to streamline the number of secondary indicators [14]. Using the hierarchical analysis method, the weight of each indicator was determined by collecting the opinions of the middle and senior management of enterprise A. According to the steps of the hierarchical analysis method, the evaluation system was successively constructed with the indicator hierarchy and the questionnaire design for two-two comparisons. The indicator screening and weight determination questionnaires were released in May 2019 and July 2019, respectively, and the questionnaire respondents were mainly from the middle and senior management of enterprise A. Finally, 11 and 7 valid questionnaires were obtained, respectively.

### 3.3. Model Construction

According to the principle of weight calculation in Yaahp software, the average weight value of each indicator was calculated. The weights of the first-level indicators (criterion level) are quality, cost, safety, time, reliability, and flexibility in descending order. It can be seen that due to the characteristics of the product category (mainly men's suits), Company A pays much attention to quality and cost.

#### 3.3.1. Data Collection

Through field research and interviews, the data of various performance evaluation indicators from 2014 to 2018 of enterprise A were summarized and normalized based on benefit-based or cost-based indicators, respectively, using different linear function methods. Subsequently, the obtained weights and the normalized data were used to calculate the total value of the supply chain performance level score for each year [15, 16]. The corresponding performance ratings were defined by the score intervals as shown in Table 1, with reference to the relevant literature and in consultation with the management of Company A.

The calculation results show that the supply chain performance level scores and performance level ratings of enterprise A from 2014 to 2018 are 0.620 (good), 0.589 (good), 0.741 (good), 0.804 (excellent), and 0.750 (excellent), respectively. The supply chain performance level of enterprise A from 2014 to 2018 shows a trend of improving year by year. On the one hand, this is the result of the comprehensive reflection of the data of 21 indicators of the enterprise; on the other hand, it is also consistent with the trend of the enterprise's business revenue and profit growth. The more objective and realistic evaluation results help to verify this result and algorithm training and play a reference role in supply chain performance improvement and optimization [17].

#### 3.3.2. Sample Training

The 5-year supply chain performance evaluation data of enterprise A are taken as the input vector matrix (*p*), and the performance evaluation levels (excellent, good, medium, and poor) in each year are taken as the target vector (*t*). The supply chain performance evaluation levels of enterprise A from 2014 to 2018 are represented by the matrix (target vector t) as follows:

(1) $$\begin{array}{c} T_{0}=\left[\begin{array}{ccccc} 0 & 0 & 0 & 0 & 0\\ 0 & 0 & 0 & 0 & 0\\ 1 & 1 & 1 & 0 & 0\\ 0 & 0 & 0 & 1 & 1 \end{array}\right]. \end{array}$$

The normalized data and the above performance matrix are placed in the same Excel sheet named “Data” and “Performance Matrix” as another expression of the input vector matrix (p), and the following code is defined in MATLAB software to associated data information.

%% BP clustering.

[adata201, bdata201, cdata201] = xlsread('LMBP performance processing data.xlsx', 'data');

[adata202, bdata202, cdata202] = xlsread('LMBP Performance Processing Data.xlsx', 'Performance Matrix');

Outputdata = adata202; %SampleClass.

Inputdata = adata201'; %sample characteristics.

Outputdata0 = Outputdata

According to the experience and related literature, the maximum training number (tp) is set to 200, the required error value (accuracy goal) is lE-9, and the learning rate (lr) is 0.1 [18]. The transfer function (*f*) between each layer is tested continuously to obtain the operation results under different function selection conditions. The mean square error reaches the minimum level of 9.02E-26 when all the interlayer functions are Tansig.

## 4. Discussion of Results

After the above discussion, the parameters of the algorithm network of the supply chain performance evaluation system of enterprise A are determined as follows: the number of nodes in the input layer is 21 (number of indicators), the number of nodes in the implicit layer is 12 (obtained experimentally), the number of nodes in the output layer is 4 (number of performance levels), the transfer function is Tansig (obtained experimentally), the training function is Trainlm (determined by the adopted algorithm), and the mean square error of the network is 9.02E-26 when the network operation is stable. Thus, the program is compiled as

Net = newff(input_train, output_train, hidnumberset(i), {'tansig', 'tansig'}, 'trainlm'); %New feedback neural network net_trainlm [19]

Where “input_train” and “output_train” point to the associated “data” and “performance The “data” is the corresponding data of 21 indicators of enterprise A for 5 years, and the “performance matrix” is To.

The training is conducted according to the parameters of classification number 1, and the output of the supply chain performance evaluation of enterprise A from 2014 to 2018 is *T.* The difference between the output of the supply chain performance evaluation Tc and the performance matrix from 2014 to 2018 is expressed as *dT*.

(2) $$\begin{array}{c} dT=10^{-13}\times \left[\begin{array}{ccccc} 0 & 0 & 0 & 0 & 0\\ 0 & 0 & 0 & 0 & 0\\ 0 & 0 & 0 & 0 & 0\\ 0 & 0 & 0 & -8.78 & -5.36\\ -1.46 & -0.20 & 0 & 0 & 0 \end{array}\right]. \end{array}$$

The five columns in the matrix represent the difference between the output results of the enterprise's performance evaluation and the performance matrix for the five years, respectively [20]. From *dT*, it can be seen that enterprise A can get more accurate performance evaluation results using this supply chain performance evaluation index system and the corresponding algorithm. This indicates that the evaluation model is reasonable and effective and can guide enterprise A to carry out supply chain performance improvement and optimization. The method can also be applied horizontally to the same type of enterprises. The relevant weight values and evaluation functions retained by the MATLAB software training process can overcome the problem of the subjective influence of professional or managerial evaluation (weight values) under the condition of the original supply chain performance level.

## 5. Some Thoughts on Performance Optimization

### 5.1. In terms of Labor Cost

Enterprise A can increase the education and training of employees, gradually improve the skills and quality of employees, and improve the labor productivity and sales performance of the enterprise by improving the skills of employees. At the same time, it can gradually introduce and strengthen the training of scientific and technological innovation talents in the fields of big data and supply chain to lay the foundation of talents for the development of the enterprise; strengthen the corporate culture propaganda to make employees realize that technological reform is an important opportunity for future development. Continuously improve the performance and salary incentive mechanisms for employees, especially compound talents, to fully motivate employees; establish a sound employee care mechanism and take a series of measures to improve employee cohesion and a sense of belonging; use big data technology to combine with the actual business operation of the enterprise to timely allocate employees and achieve optimal allocation of human resources. The above measures, if implemented, can continuously stimulate the potential of employees, which will eventually improve the business income of the enterprise and reduce the negative impact of rising labor costs and talent costs.

### 5.2. In terms of Information Technology Cost and R&D

Enterprises should fully explore the technical potential and application scope of technology software and systems introduced and start from various aspects to promote information technology to bring momentum to the growth of enterprise performance [21–23]. Offset the cost of acquiring and applying big data information by mining big data to reduce costs. For example, through information systems, we can ensure efficient, convenient, and timely delivery and sharing of product planning, technical information, training materials, product information, and customer maintenance information to effectively promote the efficient operation of each link in the supply chain and shorten the total product cycle time (lead time). From the perspective of business income, the collection, storage, processing, and analysis of customer and product-related information, effectively maintain existing customers, and improve customer loyalty, thereby promoting sales growth, while providing effective data support for the new season product planning and design, so as to more accurately develop products and services to meet consumer demand, and ultimately obtain business income growth and customer. The ultimate goal is to increase revenue and improve customer satisfaction. From the perspective of future IT cost investment, if the IT cost fluctuates significantly from year to year, it is recommended that the cost allocation should be more accurate and stable in the future, such as adopting the method of paying for software in installments for a certain period of time to ensure the stable operation of business processes.

### 5.3. In terms of Inventory Costs

Zhao believes that the use of big data analysis technology in the traditional supply chain of manufacturing can not only improve the efficiency of business operations again but also accurately compares inventory, reduce costs, and improve the efficiency of enterprise inventory management [24]. Among the specific measures, enterprises can collect, organize, record, and monitor customer (including supplier) data, sales data, weather condition data, and so on through big data technology to solve the problem of high inventory costs from three stages of the supply chain, respectively; rational planning of commodity layout in the early stage, optimizing product structure and the total number of orders placed to achieve accurate production; paying attention to goods management, display, and sales techniques in the middle stage to determine the best. In the middle stage, we pay attention to goods management, display, and sales techniques to determine the optimal amount of inventory and improve the production and sales rate of regular-priced goods; in the latter stage, we adopt a multichannel clearance mechanism to ensure a dynamic balance of low inventory.

5.4. In terms of partnership, the following measures can be taken [25, 26]. First, increase cooperation with professional enterprises such as Internet enterprises, supply chain enterprises, or logistics enterprises and establish matching outsourcing strategies; second, realize smooth information flow and sharing between the two sides with each link of the supply chain, monitor and understand the order progress through big data, and promote the smooth operation of the overall business process; furthermore, transform quantitative data into real-time visual images so that managers and operators can intuitively make or execute decisions. In addition, through historical data and big data technology, it is also possible to derive the optimal benefit distribution measures under different circumstances so that the interests of both or more parties in the cooperation can be fully guaranteed and motivated to reach the best state of cooperation and achieve synergistic development as a whole.

### 5.4. In terms of Supply Chain Flexibility

Enterprises can use the Internet and big data technology to closely link enterprises and consumers so that consumer demand data and information can be quickly communicated to producers and brand owners [27–30]. It can also organize material procurement, manufacturing, and material distribution according to changes in market demand so that the production mode can be changed from high-volume, standard-push production to market demand-pull production and closely coordinate the business processes of the supply chain to achieve more flexible management [31–39]. At the same time, it should also focus on the digitalization of product information, standardization of process technology, and multifaceted skills of production staff to improve the overall flexibility of the supply chain.

### 5.5. In terms of Supply Chain Security

Supply chain risks can be prevented and mitigated through big data technology and predictive analysis. Therefore, based on big data technology, enterprises can establish a risk control system, identify the company's business risks, and formulate corresponding preventive measures to regulate business and improve the company's risk resistance. At the level of specific measures, it can provide better support for enterprise decision-making by starting from the prerisk prediction, midoperation control, and postrisk disposal stages.

## 6. Conclusion

In order to achieve better evaluation and analysis of supply chain performance in the era of big data, this paper relies on the principle of the LMBP algorithm, supply chain performance evaluation model, and MATLAB software based on the theory of feedback neural network to conduct unified modeling and big data collection and analysis of supply chain performance of enterprise A for 5 years. The results of this paper show that the supply chain performance evaluation model based on the LMBP algorithm can provide directional suggestions for the operation management of enterprises.

## Figures and Tables

**Table 1:** An Enterprise supply chain performance score level division.

Supply chain performance level score range	Performance level
[0, 0.25]	Poor
[0.25, 0.50]	Medium
[0.50, 0.75]	Good
[0.75, 1]	Excellent

## Data Availability

The dataset can be accessed upon request.
